# Efficacy of T2∗-Weighted Gradient-Echo MRI in Early Diagnosis of Cerebral Venous Thrombosis with Unilateral Thalamic Lesion

**DOI:** 10.1155/2013/964650

**Published:** 2013-04-04

**Authors:** Shingo Mitaki, Shuhei Yamaguchi

**Affiliations:** Department of Neurology, Shimane University School of Medicine, 89-1 Enya-cho, Izumo 693-8501, Japan

## Abstract

Cerebral venous thrombosis (CVT) is an uncommon cause of stroke with diverse etiologies and varied clinical presentations. Because of variability in clinical presentation and neuroimaging, CVT remains a diagnostic challenge. Recently, some studies have highlighted the value of T2∗-weighted gradient-echo MRI (T2∗WI) in the diagnosis of CVT. We report the case of a 79-year-old woman with CVT due to a hypercoagulable state associated with cancer. On the initial T2-weighted image (T2WI), there was a diffuse high-intensity lesion in the right thalamus, extending into the posterior limb of the internal capsule and midbrain. T2∗WI showed diminished signal and enlargement of the right basilar vein and the vein of Galen. Even though there is a wide range of differential diagnoses in unilateral thalamic lesions, and a single thalamus lesion is a rare entity of CVT, based on T2∗WI findings we could make an early diagnosis and perform treatment. Our case report suggests that T2∗WI could detect thrombosed veins and be a useful method of early diagnosis in CVT.

## 1. Introduction

Cerebral venous thrombosis (CVT) is an uncommon cause of stroke with diverse etiologies and varied clinical presentations. The diagnosis of CVT is based on neuroimaging. However, in contrast to arterial strokes, brain imaging by itself is of little positive value for the diagnosis of CVT because it can be normal in up to 25% of patients [1]. Because of variability in clinical presentation and neuroimaging, delays in diagnosis of CVT are common, despite early diagnosis being essential; 10% of CVT patients are found to have permanent neurological deficits by 12 months of followup [2]. 

Recently, Selim et al. have highlighted the value of T2*WI, which show the thrombosis as a hypointense signal associated with the magnetic susceptibility effect [3, 4]. They have reported that the T2*-weighted MR sequence can be useful in rapid detection of CVT and may enable the diagnosis to be made prior to MR venography (MRV) [3]. 

Here, we describe a rare case of CVT with unilateral thalamic lesion in which we could detect the thrombosed veins with T2*WI. This finding led us to the early diagnosis of CVT.

## 2. Case Report

A 79-year-old woman presented to the emergency department with complaints of weakness and numbness in the left upper and lower extremities. She had been diagnosed with gastric cancer (adenocarcinoma of the gastric cardia, T2N3 M0) 5 years previously, and chemo- and radiotherapy were performed at that time. However, her cancer was progressive and she had developed stenosis of the cardiac portion of the esophagus.

Neurological examination revealed left-sided hypoesthesia, and left-sided hemiparesis with a National Institutes of Health Stroke Scale (NIHSS) score of 7. Although the patient did not fulfill the diagnostic criteria of disseminated intravascular coagulation syndrome, she had abnormal coagulation with a fibrinogen level of 191 mg/dL; fibrin/fibrinogen degeneration products, 47.9 *μ*g/mL; and D dimer, 29.2 *μ*g/mL. The other laboratory parameters, including protein C, S, antinuclear antibody, antiphospholipid antibody, and homocysteine, were within normal limits. 

**Figure 1:**
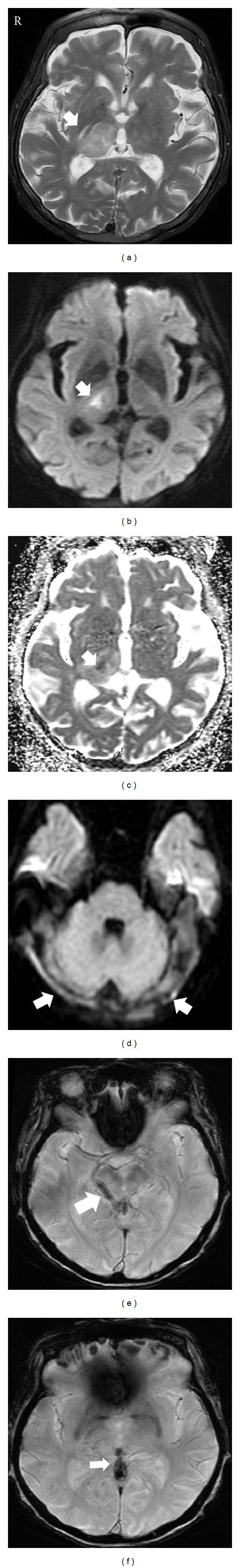
On admission, T2-weighted images (T2WIs) showed a diffuse high-intensity lesion in the right thalamus, extending into the posterior limb of the internal capsule and midbrain (a). The central part of the right thalamic lesion showed high intensity on the diffusion-weighted image, (b) and a decreased apparent diffusion coefficient (c). Both transverse sinuses showed high intensity on the diffusion-weighted image (d). T2*WI showed diminished signal and enlargement of the right basilar vein (e) and the vein of Galen (f).

**Figure 2:**
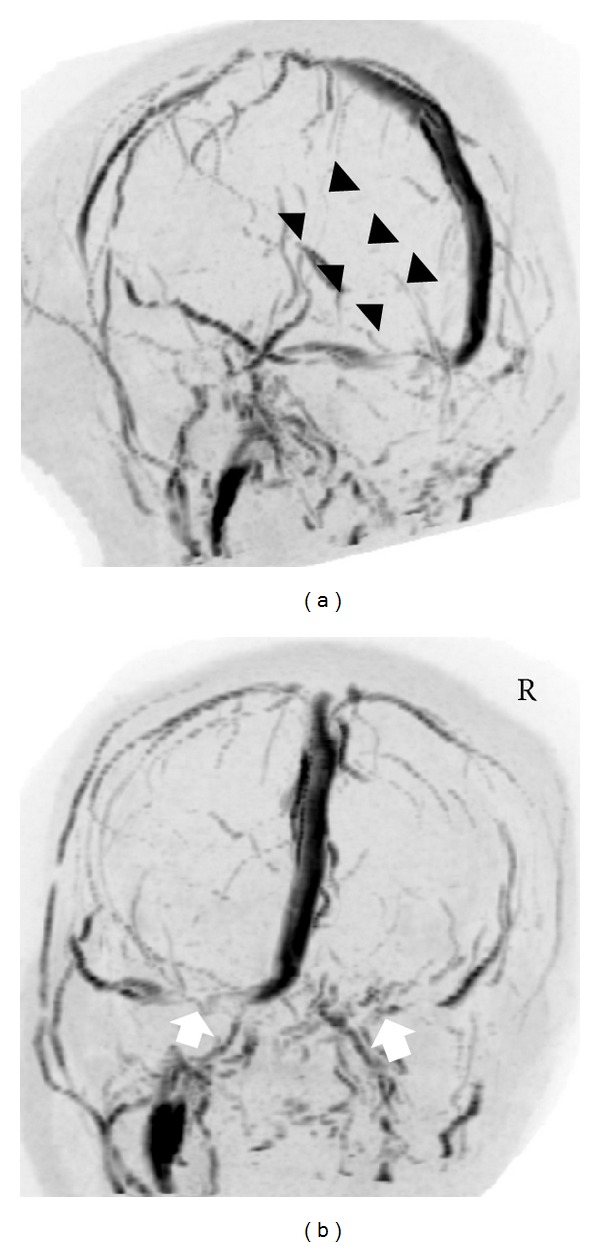
Two days after admission, magnetic resonance venography (MRV) showed an absence of flow in the inferior sagittal sinus, vein of Galen, and straight sinus (a), and a reduction of flow in the transverse sinus (b).

On the initial T2WI, there was a diffuse high-intensity lesion in the right thalamus, extending into the posterior limb of the internal capsule and midbrain (Figure 1(a)). The central part of the right thalamic lesion showed high intensity on the diffusion-weighted image (DWI) (Figure 1(b)), and a decreased apparent diffusion coefficient (Figure 1(c)). Both transverse sinuses also showed high intensity on the DWI (Figure 1(d)). The T2*WI showed diminished signal and enlargement of the right basilar vein (Figure 1(e)) and the vein of Galen (Figure 1(f)). On the basis of the MRI findings, she was diagnosed with CVT, and anticoagulation therapy with heparin was administered. Two days later, MRV showed an absence of flow in the inferior sagittal sinus, vein of Galen, and straight sinus (Figure 2(a)), and a reduction of flow in the transverse sinus (Figure 2(b)).

## 3. Discussion

The novel finding in our patient is that T2*WI was a useful tool for the early diagnosis of CVT. Moreover, our case demonstrated that T2*WI could detect thrombosed veins. Recent report has shown that a T2*-weighted gradient-echo MR sequence has more sensitivity than spin-echo T1-weighted image (T1WI), T2WI, and fluid-attenuated inversion recovery images (FLAIR) in detecting CVT, as well as in detecting subarachnoid and intracerebral hemorrhages, both of which can be seen in association with CVT [3]. Thrombosed veins were recently detected on T2*WI as a loss of signal resulting from the susceptibility effects of deoxyhemoglobin within the blood clots; this occurs as red thrombi at the site of venous occlusion [3]. Because susceptibility effects are most pronounced in venous segments with more acute thrombi, application of a T2*-weighted gradient-echo MR sequence may be useful during this stage for diagnosis [5]. Furthermore, acute-stage venous thrombus is difficult to recognize on MRI [6] because of its typical signal intensity (isointense or hypointense on T1WI and hypointense on T2WI). Therefore, T2*WI could play an important role in the early detection of CVT.

Thalamic edema is the imaging hallmark of deep venous occlusion, as demonstrated by hypersignal intensity on the T2WI and FLAIR [7]. Deep venous infarction is usually symmetrical, affecting the thalamus bilaterally [7]. In contrast, CVT that only affects the thalamus unilaterally, as observed in our patient, is a rare entity [8]. Because there is a wide range of differential diagnoses in unilateral thalamic lesions, it can be expected that additional time is required to make a definitive diagnosis, which could result in treatment delay. Our case suggests that particular imaging techniques, including T2*WI, are helpful in establishing the correct diagnosis. 

As shown in Figure 1, high intensity in the transverse sinus on DWIs also suggests the presence of a thrombus, which was confirmed by MRV. DWI of intravascular clots has been recently reported in the literature [9]. A hyperintense signal involving sinuses or veins on the DWI was detected in 41% of cases with CVT [9]. Although, in our case, the DWI was also useful for early detection of CVT, this was reported not to be superior to conventional MRI for the acute detection of clots in CVT [9]. Further studies on the importance of DWI for early diagnosis of CVT are warranted. 

Previously, it was reported that cancer and tumors accounted for 7.4% of all CVT. Of these, 2.2% were associated with CNS malignancy, 3.2% with solid tumors outside the CNS, and 2.9% with hematological disorders [10]. In our case, the hypercoagulable state that accompanies cancer is thought to be a causal factor for CVT. Since cancer is a predictor of death or dependence in CVT patients [10], in case CVT is diagnosed, the possibility of a paraneoplastic syndrome should be considered. 

In conclusion, even though a patient presented with a rare case of unilateral thalamic lesions, assessment of T2*WI was a useful method of early diagnosis in CVT. Further studies on the usefulness of T2*WI in the evaluation of CVT are warranted.

## References

[1] Bousser M-G, Ferro JM (2007). Cerebral venous thrombosis: an update. *The Lancet Neurology*.

[2] Dentali F, Gianni M, Crowther MA, Ageno W (2006). Natural history of cerebral vein thrombosis: a systematic review. *Blood*.

[3] Selim M, Fink J, Linfante I, Kumar S, Schlaug G, Caplan LR (2002). Diagnosis of cerebral venous thrombosis with echo-planar T2*-weighted magnetic resonance imaging. *Archives of Neurology*.

[4] Idbaih A, Boukobza M, Crassard I, Porcher R, Bousser MG, Chabriat H (2006). MRI of clot in cerebral venous thrombosis: high diagnostic value of susceptibility-weighted images. *Stroke*.

[5] Leach JL, Strub WM, Gaskill-Shipley MF (2007). Cerebral venous thrombus signal intensity and susceptibility effects on gradient recalled-echo MR imaging. *American Journal of Neuroradiology*.

[6] Hinman JM, Provenzale JM (2002). Hypointense thrombus on T2-weighted MR imaging: a potential pitfall in the diagnosis of dural sinus thrombosis. *European Journal of Radiology*.

[7] Crombé D, Haven F, Gille M (2003). Isolated deep cerebral venous thrombosis diagnosed on CT and MR imaging. A case study and literature review. *Journal Belge de Radiologie*.

[8] Herrmann KA, Sporer B, Yousry TA (2004). Thrombosis of the internal cerebral vein associated with transient unilateral thalamic edema: a case report and review of the literature. *American Journal of Neuroradiology*.

[9] Favrole P, Guichard JP, Crassard I, Bousser MG, Chabriat H (2004). Diffusion-weighted imaging of intravascular clots in cerebral venous thrombosis. *Stroke*.

[10] Ferro JM, Canhão P, Stam J, Bousser MG, Barinagarrementeria F (2004). Prognosis of cerebral vein and dural sinus thrombosis: results of the International Study on Cerebral Vein and Dural Sinus Thrombosis (ISCVT). *Stroke*.

